# Lost before found: A new species of whaler shark *Carcharhinus obsolerus* from the Western Central Pacific known only from historic records

**DOI:** 10.1371/journal.pone.0209387

**Published:** 2019-01-02

**Authors:** William T. White, Peter M. Kyne, Mark Harris

**Affiliations:** 1 CSIRO Australian National Fish Collection, National Research Collections Australia, Hobart, Tasmania, Australia; 2 Research Institute for the Environment and Livelihoods, Charles Darwin University, Darwin, Northern Territory, Australia; 3 F.F.C. Elasmobranch Studies, New Port Richey, Florida, United States of America; Ecole Normale Supérieure de Lyon, FRANCE

## Abstract

*Carcharhinus obsolerus* is described based on three specimens from Borneo, Thailand and Vietnam in the Western Central Pacific. It belongs to the *porosus* subgroup which is characterised by having the second dorsal-fin insertion opposite the anal-fin midbase. It most closely resembles *C*. *borneensis* but differs in tooth morphology and counts and a number of morphological characters, including lack of enlarged hyomandibular pores which are diagnostic of *C*. *borneensis*. The historic range of *C*. *obsolerus* sp. nov. is under intense fishing pressure and this species has not been recorded anywhere in over 80 years. There is an urgent need to assess its extinction risk status for the IUCN Red List of Threatened Species. With so few known records, there is a possibility that *Carcharhinus obsolerus* sp. nov. has been lost from the marine environment before any understanding could be gained of its full historic distribution, biology, ecosystem role, and importance in local fisheries.

## Introduction

The life sciences rely on a strong taxonomic underpinning of the faunal groups being investigated, without which it is extremely difficult to put such research into the correct context [1, 2]. However, the discovery of species has infinite possibilities, and it is not always possible to predict where taxonomic discoveries or problems lie. Chondrichthyan fishes (sharks, rays and chimaeras) are a prime example where in recent decades, taxonomy of this group has undergone somewhat of a renaissance. More than 180 new chondrichthyan species were formally described between 2002 and 2012 [2], with more than 80 new species formally described since 2012. Thus, more than 20% of the extant species of sharks and rays have been described since 2002. The discovery of species new to science are made in a number of ways, ranging from exploration into deeper waters and more remote locations, e.g. [3, 4], to detailed taxonomic revisions of specific groups yielding higher diversity than previously thought, e.g. [5, 6]. Add to this, the rapidly growing area of genetics which in recent years has provided a useful tool to taxonomists and enabled the far more rapid identification of potential species complexes and cryptic species [2].

Several chondrichthyan groups have recently undergone relatively large expansions in their diversity due to taxonomic studies revealing previously unknown species. A prime example of this is in the Neotropical stingray family Potamotrygonidae. Of the 34 valid nominal species in this family, 14 were described since 2006 with seven of these since 2013 [7]. Similarly, of the 35 species in the dogfish family Squalidae, 19 of these were described since 2007 [8–10], with a further two resurrected as valid species since that year. In contrast, some recent taxonomic studies have reduced the number of valid taxa. For example, the manta and devilrays (family Mobulidae) were previously considered to consist of two genera and 11 species, but a recent study found they consist of only a single genera and 8 valid species [11].

The whaler sharks, family Carcharhinidae, are one of the most economically important groups of sharks in fisheries globally, particularly in tropical regions, in commercial and small-scale coastal fisheries. Thus, it is imperative to have a strong taxonomic foundation from which to work from, both in terms of conservation efforts and also fisheries management. The most speciose genus is *Carcharhinus* Blainville which consists of 35 of the 57 carcharhinid species. In general, the taxonomy of this genus has been relatively stable with only 11 species described since 1905, and all but one described before 1986. One exception is the *dussumieri-sealei* group where recent taxonomic investigation [12, 13] resulted in description of a new species, *Carcharhinus humani* White & Weigmann, and the resurrection of two species previously not considered valid, *Carcharhinus coatesi* (Whitley) and *C*. *tjutjot* (Bleeker). Another species was also recently resurrected, *Carcharhinus cerdale* Gilbert [14], which was previously considered synonymous with *C*. *porosus* (Ranzani).

One species which has remained unresolved is the *Carcharhinus* sp. first listed in [15, 16], and subsequently as *Carcharhinus* sp. A in [17] and [18], initially based on a single specimen from Borneo [15], with two additional specimens subsequently recognised from Vietnam and Thailand [16]. These three specimens were discussed in detail by [19] in his excellent revision of the genus *Carcharhinus*. In this study, Garrick tentatively referred these specimens to *Carcharhinus porosus*, but noted a number of subtle morphometric differences. It was considered an undescribed species by [16] based on cranial differences and morphometrics, but without specific details provided. *Carcharhinus* sp. A was considered a species in question by [18] and they provided descriptive information, largely adapted from [19]. These authors concluded that this species should be considered as conspecific with *C*. *porosus* from the Americas, despite the improbable distribution pattern. Recent examination of the three specimens of *Carcharhinus* sp. A by one of us (WW) revealed that they should not be considered conspecific with *C*. *porosus* from the Western Atlantic because they differ from it in dentition and several other morphological characters. One of the main reasons this species has remained largely unresolved is that two of the known specimens are juveniles and the third a late-term embryo. While examination of additional specimens, particularly adults, would be prudent, no additional specimens have been found in collections or during any of the numerous surveys conducted in its known range since it was first flagged as a potentially undescribed species. The uncertain status of this small, presumably coastal species in the heavily fished South-east Asian region made a thorough investigation of its taxonomy a priority. Based on the results of this investigation, these three specimens are formally named and described as a new species and comparisons are made with its congeners.

## Materials and methods

### Ethics statement

The specimens upon which the new species is based were collected over 80 years ago and are deposited in the Academy of Natural Sciences, Philadelphia (ANSP) and the Naturhistorisches Museum, Vienna (NMW). Permission to examine them was obtained from both museums, and two ANSP specimens were formally loaned to the Australian National Fish Collection (CSIRO). No new material was collected for this study.

### Comparative material

Specimens of closely related congeneric taxa examined for comparison were (see Acknowledgments for institutional names): *Carcharhinus borneensis* (Bleeker) (2 RMNH, 3 CSIRO, 7 IPPS); *C*. *cerdale* (3 CAS, 2 BMNH); *C*. *macloti* (Müller & Henle) (6 CSIRO, 7 KFRS). A list of all comparative specimens used in this study are listed below. Collection abbreviations are: Natural History Museum, London, U.K. (BMNH); Stanford University Collection, housed at Californian Academy of Sciences, San Francisco, USA (CAS-SU); CSIRO Australian National Fish Collection, Hobart, Australia (CSIRO); Kanudi Fisheries Research Station, housed at University of PNG, Port Moresby (KFRS); Jason C. Seitz personal collection, USA (JCS); Naturhistorisches Museum, Vienna, Austria (NMW); Mark Harris private collection, Florida, USA (PMH); and Naturalis—National Natuurhistorisch Museum, Leiden, Netherlands (RMNH).

#### Carcharhinus borneensis

RMNH 7386 (holotype), immature male 238 mm TL (fresh umbilical scar present), Singkawang, West Kalimantan, Indonesia; CSIRO H 6226–01, juvenile male 341 mm TL, CSIRO H 6226–02, female 348 mm TL, Mukah, Sarawak, Malaysia, 02°53.52’ N, 112°05.44’ E, 8 Apr. 2004; CSIRO H 6212–01, adult male 576 mm TL, Mukah, Sarawak, Malaysia, 02°53.52’ N, 112°05.44’ E, 27 Apr. 2004; PMH244-1 (dried jaws), mature female 661 mm TL, PMH244-2 (dried jaws), mature female 634 mm TL, Mukah fish landings, Sarawak, Malaysia, 7 Jul. 2004; PMH244-3 (dried jaws), mature male 595 mm TL, Mukah fish landings, Sarawak, Malaysia, 18 Jul. 2004; PMH244-4 (dried jaws), mature male 579 mm TL, Mukah fish landings, Sarawak, Malaysia, 16 Jul. 2004.

#### Carcharhinus cerdale

CAS-SU 11884 (holotype), juvenile male 559 mm TL, Panama; BMNH 1903.5.15.339.40 (paratypes; 2 specimens), adult male 889 mm TL, juvenile male 675 mm TL, Panama; CAS-SU 12865 (paratype), juvenile male 525 mm TL, Panama; CAS-SU 11886 (paratype), female 604 mm TL, Panama; CAS-SU 11886 (paratype), juvenile male 559 mm TL, Panama; NMW 61348, juvenile male 675 mm TL, Panama; PMH329-01 (dried jaws), immature male 887 mm TL, Puerto Caimito, Panama, 15 May 1999.

#### Carcharhinus macloti

CSIRO H 7834–01, juvenile male 410 mm TL, south of Deception Bay, Gulf of Papua, 8°1’54” S, 144°39’13” E, 22–25 m depth, 15 Dec. 2014; CSIRO H 7835–01, juvenile male 410 mm TL, south of Deception Bay, Gulf of Papua, 8°2’16” S, 144°38’26” E, 22–23 m depth, 15 Dec. 2014; CSIRO H 7825–02 (dried jaw), female 840 mm TL, east of Fly River mouth, Gulf of Papua, 8°37’ S, 144°11’ E, 17–19 m depth, 13 Dec. 2014; CSIRO H 8153–01 (dried jaw), adult male 770 mm TL, east of Fly River mouth, Gulf of Papua, 8°36’48” S, 144°11’19” E, 17–23 m depth, 1 Apr. 2015; CSIRO H 8108–01 (dried jaw and cranium), female 810 mm TL, south of Kerema, Gulf of Papua, 8°1’58.08” S, 145°45’17.52” E, 12–15 m depth, 16 Sep. 2015; CSIRO H 8152–02 (dried jaw and cranium), female 820 mm TL, south of Deception Bay, Gulf of Papua, 8°9’ S, 144°28’ E, 19–23 m depth, 9 Dec. 2014; KFRS E.751, juvenile male 390 mm TL, Freshwater Bay, Gulf of Papua, 8°11’ S, 146°1’ E, 19–20 m depth, 29 Nov. 2014; PMH057-07 (dried jaws), mature female 925 mm TL, Tuticorin Harbor fish market, Thoothukudi, Tamil Nadu, India, 10 Nov. 2001; PMH057-09 (dried jaws), mature female 910 mm TL, Tuticorin Harbor fish market, Thoothukudi, Tamil Nadu, India, 13 Nov. 2001; PMH057-10 (dried jaws), mature female 904 mm TL, Tuticorin Harbor fish market, Thoothukudi, Tamil Nadu, India, 10 Nov. 2001; PMH057-14 (dried jaws), mature male 970 mm TL, Dong Gang fish market, Kaohsiung, Taiwan, 26 Sep. 2003; PMH057-15 (dried jaws), mature female 1090 mm TL, Dong Gang fish market, Kaohsiung, Taiwan, 10 Aug. 2003; PMH057-19 (dried jaws), mature female 993 mm TL, Kudat fish landings, Sabah, Malaysia, 14 Apr. 2004; PMH057-21 (dried jaws), mature male 980 mm TL, Kudat fish landings, Sabah, Malaysia, 6 Jun. 2005; PMH057-27 (dried jaws), mature female 920 mm TL, Pasil fish port, Cebu City, Philippines, 23 May 2010; PMH057-28 (dried jaws), mature male 930 mm TL, Pasil fish port, Cebu City, Philippines, 19 Apr. 2010; PMH057-33 (dried jaws), immature female 643 mm TL, Dong Gang fish market, Kaohsiung, Taiwan, 10 Aug. 2003; PMH057-37 (dried jaws), mature female 1020 mm TL, off Nha Trang, Vietnam, 26 Oct. 2007.

#### Carcharhinus porosus

PMH121-01 (dried jaws), mature female, 1085 mm TL, Manzanilla Bay, Trinidad, 17 Aug. 1985; PMH121-02 (dried jaws), mature female, 996 mm TL, Manzanilla Bay, Trinidad, 8 Aug. 1985; JCS CP241107 (dried jaws), female 987 mm TL, Manzanilla Bay, Trinidad, 8 Aug. 1985.

### Morphology

External morphometric measurements were taken by digital vernier calipers to one tenth of a millimetre (mm) from specimens preserved in 70% ethanol. Measurement terminology follows [16, 20] except for total length (TL) and additional direct (point-to-point) measurements which were taken, i.e. pre-first-dorsal-fin-length (PD1), head length (HDL), prebranchial length (PG1), preorbital length (POB), prenarial snout length (PRN). Direct measurements are used in the description if not otherwise stated. Fin origin was deemed to be the point of greatest angle (as used by [19]). Note that in [21] for *C*. *borneensis*, the fin origins, particularly those of the dorsal and anal fins, were determined to be further forward on the body, at the start of the short ridge before these fins. This explains the differences between the length, base length and anterior margin of the first dorsal, second dorsal and anal fins reported in [21] and our data. Dentitional terms follow [16, 19, 20]. Cranial morphology follows [16] and is based on radiograph of the head of one of the paratypes (ANSP 77121). The holotype and paratypes of the new species were measured in full (Table 1). In the description and diagnosis, values for the holotype are given first, followed by the ranges for the paratypes in parantheses.

**Table 1:** Morphometric data for *Carcharhinus obsolerus*. Morphometric data for the holotype (NMW 61463) and two paratypes. Measurements expressed as a percentage of total length; D1 refers to first dorsal fin, D2 to second dorsal fin.

	Holotype	Paratype	Paratype	Types combined	
	NMW 61463	ANSP 77121	ANSP 76859	Min.	Max.
Total length (mm)	433	370	339	339	433
Fork length	82.0	78.9	99.1	78.9	99.1
Precaudal length	74.4	71.2	70.2	70.2	74.4
Pre-second dorsal length	62.8	60.0	59.7	59.7	62.8
Pre-first dorsal length	32.5	27.7	28.7	27.7	32.5
Head length	24.7	25.1	23.9	23.9	25.1
Head length (horizontal)	24.2	23.9	22.6	22.6	24.2
Prebranchial length	20.6	20.9	19.5	19.5	20.9
Prebranchial length (horizontal)	20.6	20.9	19.5	19.5	20.9
Preorbital length	9.9	9.9	9.0	9.0	9.9
Preorbital length (horizontal)	8.3	8.7	7.7	7.7	8.7
Preoral length	8.9	9.2	8.0	8.0	9.2
Snout to nostril	6.4	6.7	5.5	5.5	6.7
Prenarial length (horizontal)	6.4	6.7	5.5	5.5	6.7
Prepectoral length	23.2	24.6	21.7	21.7	24.6
Prepelvic length	48.5	47.0	44.5	44.5	48.5
Snout-vent length	50.1	49.2	47.2	47.2	50.1
Preanal length	60.7	59.5	57.5	57.5	60.7
Interdorsal space	22.4	21.4	22.0	21.4	22.4
Dorsal-caudal space	8.0	6.3	6.7	6.3	8.0
Pectoral-pelvic space	18.8	19.2	18.3	18.3	19.2
Pelvic-anal space	7.7	8.5	8.4	7.7	8.5
Anal-caudal space	8.5	6.5	6.6	6.5	8.5
Eye length	2.4	2.5	2.9	2.4	2.9
Eye height	2.5	2.4	2.7	2.4	2.7
Interorbital space	11.9	12.0	11.2	11.2	12.0
Nostril width	2.4	1.9	1.7	1.7	2.4
Internarial space	5.9	6.6	6.2	5.9	6.6
Anterior nasal flap length	0.5	0.6	0.6	0.5	0.6
Mouth length	5.0	5.3	4.9	4.9	5.3
Mouth width	8.3	8.8	8.9	8.3	8.9
Upper labial furrow length	0.3	0.6	0.5	0.3	0.6
Lower labial furrow length	0.0	0.0	0.0	0.0	0.0
First gill slit height	2.6	2.1	2.6	2.1	2.6
Third gill slit height	3.3	2.3	2.9	2.3	3.3
Fifth gill slit height	2.4	1.9	2.3	1.9	2.4
Intergill distance	4.4	4.6	4.2	4.2	4.6
Head height	11.2	10.7	11.8	10.7	11.8
TRH-Trunk height	12.5	12.8	13.0	12.5	13.0
Abdomen height	11.2	11.4	-	11.2	11.4
Tail height	8.4	8.6	9.5	8.4	9.5
Caudal peduncle height	3.8	4.3	4.7	3.8	4.7
Head width	11.6	12.1	13.4	11.6	13.4
Trunk width	10.6	10.6	12.7	10.6	12.7
Abdomen width	9.8	9.3	-	9.3	9.8
Tail width	7.1	7.1	7.9	7.1	7.9
Caudal peduncle width	3.6	3.7	3.3	3.3	3.7
Pectoral-fin length	10.7	11.6	11.0	10.7	11.6
Pectoral-fin anterior margin	14.6	16.8	15.2	14.6	16.8
Pectoral-fin base length	6.1	6.9	6.1	6.1	6.9
Pectoral-fin height	12.7	13.7	12.4	12.4	13.7
Pectoral-fin inner margin	5.2	5.5	5.5	5.2	5.5
Pectoral-fin posterior margin	11.0	12.2	10.9	10.9	12.2
Pelvic-fin length	7.5	7.7	8.1	7.5	8.1
Pelvic-fin anterior margin	5.4	5.7	5.6	5.4	5.7
Pelvic-fin base length	4.3	4.8	4.8	4.3	4.8
Pelvic-fin height	4.4	4.9	4.6	4.4	4.9
Pelvic-fin inner margin	3.3	3.5	3.2	3.2	3.5
Pelvic-fin posterior margin	5.2	5.3	5.6	5.2	5.6
D1 length	13.7	17.0	16.1	13.7	17.0
D1 anterior margin	10.9	13.7	13.4	10.9	13.7
D1 base length	10.0	11.2	11.2	10.0	11.2
D1 height	8.2	9.9	8.3	8.2	9.9
D1 inner margin	4.0	5.9	4.4	4.0	5.9
D1 posterior margin	10.0	11.7	9.7	9.7	11.7
D2 length	7.5	8.0	6.9	6.9	8.0
D2 anterior margin	4.2	4.0	3.4	3.4	4.2
D2 base length	4.0	4.0	3.6	3.6	4.0
D2 height	2.0	2.2	2.6	2.0	2.6
D2 inner margin	3.4	4.4	3.3	3.3	4.4
D2 posterior margin	4.3	5.0	4.4	4.3	5.0
Anal-fin length	8.1	8.5	8.2	8.1	8.5
Anal-fin anterior margin	6.1	5.5	5.3	5.3	6.1
Anal-fin base	4.8	4.5	4.3	4.3	4.8
Anal-fin height	2.9	2.8	3.1	2.8	3.1
Anal-fin inner margin	3.5	4.2	3.9	3.5	4.2
Anal-fin posterior margin	3.4	4.9	4.1	3.4	4.9
Dorsal caudal margin	25.0	28.1	29.9	25.0	29.9
Preventral caudal margin	11.0	11.9	11.2	11.0	11.9
Lower postventral caudal margin	4.7	5.9	5.0	4.7	5.9
Upper postventral caudal margin	11.9	15.5	15.0	11.9	15.5
Caudal fork width	6.4	7.5	7.5	6.4	7.5
Caudal fork length	9.1	9.0	9.4	9.0	9.4
Subterminal caudal margin	3.8	3.3	3.0	3.0	3.8
Subterminal caudal width	2.9	3.2	3.1	2.9	3.2
Terminal caudal margin	5.9	7.3	6.4	5.9	7.3
Terminal caudal lobe	7.9	8.8	8.3	7.9	8.8
D2 origin to anal-fin origin	2.5	1.3	1.9	1.3	2.5
D2 insertion to anal-fin insertion	1.3	0.8	1.0	0.8	1.3
D1 midpoint to pectoral-fin insertion	6.7	5.0	6.4	5.0	6.7
D1 midpoint to pelvic-fin origin	11.5	13.4	12.4	11.5	13.4
Pelvic-fin midpoint to D1 insertion	9.5	10.5	8.9	8.9	10.5
Pelvic-fin midpoint to D2 origin	12.1	11.0	12.0	11.0	12.1

### Meristics

Vertebral terminology, method of counting and vertebral ratios follow [16, 20, 22]. Meristics were taken from radiographs of the type specimens. Counts were obtained separately for trunk (monospondylous), precaudal (monospondylous + diplospondylous to origin of upper lobe of caudal fin) and caudal (centra of the caudal fin) vertebrae. Tooth row counts were made in situ.

To enable comparisons with congeners, precaudal and total vertebral counts, and upper and lower tooth counts for all valid species of *Carcharhinus* were collated from the following sources: [12, 19, 21, 23–29]. These data, including counts for the new species, are presented in Table 1. The species groups used generally follow [16] with some modification based on more recent molecular and taxonomic studies, e.g. [12, 13, 30].

### Nomenclatural acts

The electronic edition of this article conforms to the requirements of the amended International Code of Zoological Nomenclature, and hence the new name contained herein is available under that Code from the electronic edition of this article. This published work and the nomenclatural act it contains have been registered in ZooBank, the online registration system for the ICZN. The ZooBank LSID (Life Science Identifiers) can be resolved and the associated information viewed through any standard web browser by appending the LSID to the prefix “http://zoobank.org/”. The LSID for this publication is: urn:lsid:zoobank.org:pub: ADB5D2F7-3D61-4085-8869-5BF815D9DAD5. The electronic edition of this work was published in a journal with an ISSN, and has been archived and is available from the following digital repositories: PubMed Central, LOCKSS.

## Results

### Genus *Carcharhinus* Blainville

Type species. *Carcharias melanopterus* Quoy & Gaimard, under suspension of the Rules by the ICZN [31].

#### Definition

Adapted from [19]: Small to large carcharhinids with the following combination of characters: an internal nictitating lower eyelid; no spiracles (rarely present in juveniles as minute vestiges); short labial furrows, their length less than 1% TL, the lower barely or not visible when mouth is closed; snout short to moderately long, preoral length always less than 10% TL; internarial distance at least 2.5 times nostril width; teeth blade-like with single cusps, although basal margins of cusps may have enlarged serrae; cusps of upper teeth serrated or smooth; total number files of teeth in upper or lower jaws less than 40; midpoint of first dorsal-fin base usually closer, or at least as close to, pectoral-fin free tip than to pelvic-fin origin; height of second dorsal fin never more than 55% height of first dorsal fin, 60–120% of height of anal fin; second dorsal fin more or less opposite anal fin, its origin usually in front of midpoint of anal-fin base, but rarely over posterior third of anal-fin base; upper and lower precaudal pits present, upper better developed, crescent shaped, wider than long, with a well-defined anterior edge; caudal peduncle without lateral dermal ridges.

### New species description

*Carcharhinus obsolerus* White, Kyne & Harris **sp. nov.** urn:lsid:zoobank.org:act:DCE2AA0F-1C75-4849-B572-4FD5472D57D1.

#### Synonymy

*Carcharhinus* sp.: [15]: 517, 520, 523, 536 (Borneo); [16]: 319, 321, 327 (Vietnam, Borneo, and Thailand); [32]: 1359, fig (Vietnam, Borneo, and Thailand)

*Carcharhinus porosus*: [19]: 71 (Borneo, Saigon, and Bangkok)

*Carcharhinus* undescribed small species: [33]: 497 (Borneo, Vietnam, and Thailand)

*Carcharhinus* sp. (= ‘*Carcharhinus porosus*’): [32]: 1322.

*Carcharhinus* sp. A: [17]: 307, fig, pl. 62 (Borneo, Vietnam, and Thailand); [18]: 103, fig 50

#### Holotype

NMW 61463, female 433 mm TL, Bangkok, Thailand, no date or collector recorded.

#### Paratypes

ANSP 76859, female late-term embryo 339 mm TL, Ho Chi Minh City, Vietnam, Dec. 1934, coll. H. Rutherfurd; ANSP 77121 (paratype of *Carcharhinus tephrodes* Fowler), female 370 mm TL, Baram, Sarawak, Malaysian Borneo, 1897, coll. A.C. Harrison Jr. & H.M. Hiller.

#### Diagnosis

A small species of *Carcharhinus* with: a slender body and tail; no interdorsal ridge; head parabolic in dorsal view, relatively wide, interorbital space 11.2–12.0% TL; eyes relatively large, length 2.4–2.9% TL, 10.0–15.1 times in head length; no row of enlarged hyomandibular pores alongside each mouth corner; upper anterior teeth broadly triangular and serrated, with large and coarse (non-lobate) serrations basally; lower anterior teeth with narrower, mostly straight cusps; cusps of upper and lower anterolateral teeth with apical margin slightly recurved; no lateral cusplets; total tooth row counts 27–31/26–29; posterior edge of the mandibular plate with an elongate and crescentic indentation; second dorsal-fin origin well posterior of anal-fin origin, about opposite anal-fin midbase, second dorsal-fin origin to anal-fin origin 1.3–2.5% TL, 0.3–0.6 times second dorsal-fin base; first dorsal fin triangular, not falcate, origin about opposite first third of pectoral-fin inner margin length, free rear tip just anterior to pelvic-fin origins, length 1.7–1.9 times height, inner margin 1.9–2.5 in base; second dorsal fin much smaller than first, slightly smaller than anal fin; base 1.4–2.0 times height; height 22–31% of first dorsal-fin height; anal fin height 1.2–1.5 times second dorsal height, base 1.1–1.2 times second dorsal-fin base; total vertebral counts 114–120, monospondylous precaudal counts 36–40, diplospondylous precaudal counts 18–19, diplospondylous caudal counts 56–66, precaudal counts 54–58; no distinct black markings on fins.

#### Description

**Fig 1:**
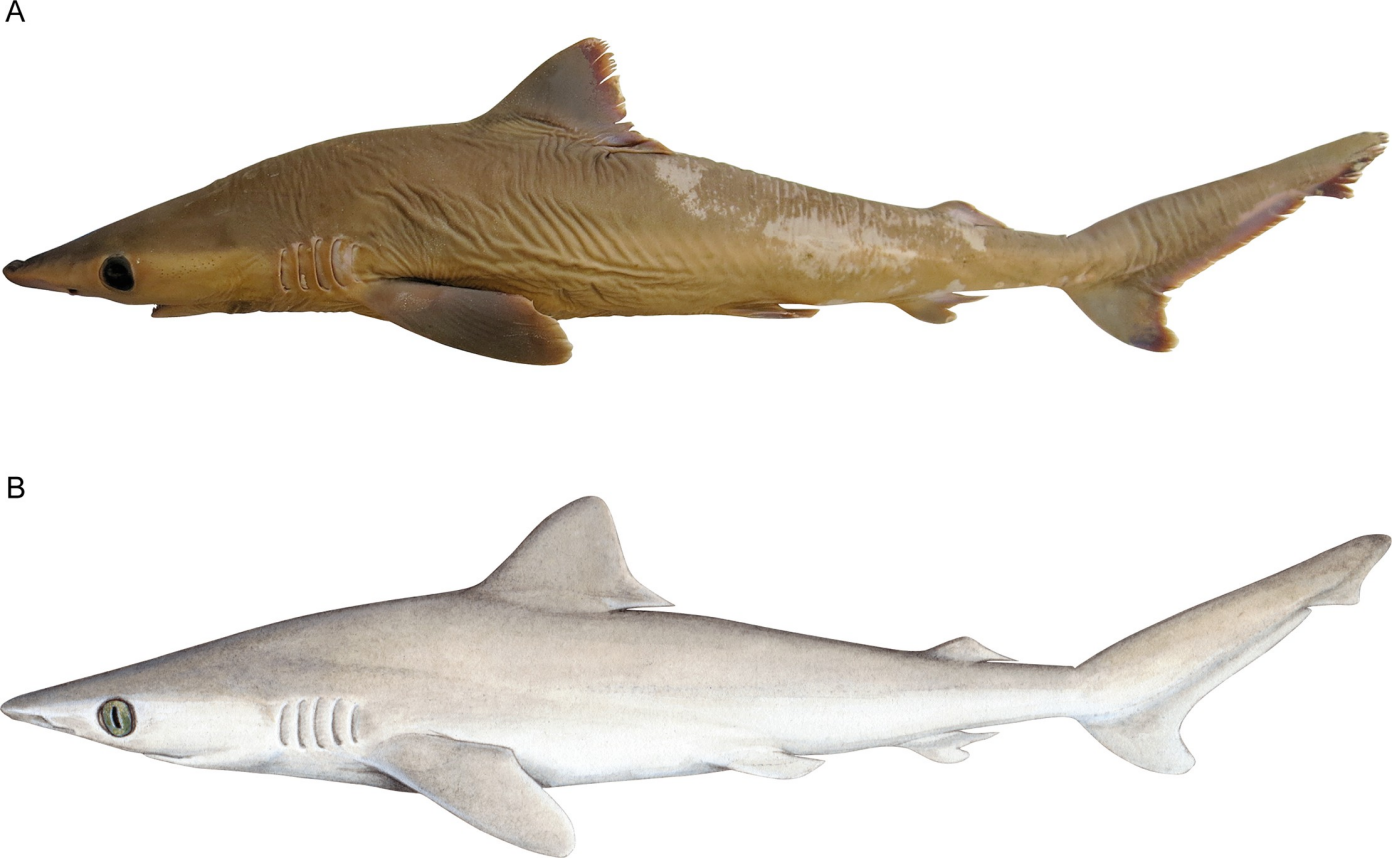
Lateral view of *Carcharhinus obsolerus* sp. nov. (NMW 61463; female holotype 433 mm TL). A. Preserved specimen; B. Painting by Lindsay Marshall (www.stickfigurefish.com.au).

Body moderately slender (Fig 1), trunk subcircular and almost pear-shaped in section at first dorsal-fin base, length of trunk from fifth gill slits to vent 1.07 in holotype (1.06–1.09 in paratypes) times head length. Predorsal, interdorsal and postdorsal ridges absent from midline of back, lateral ridges absent from body. Caudal peduncle relatively slender, rounded-hexagonal in section at second dorsal-fin insertion, postdorsal and postventral spaces flattened, lateral surfaces subangular; height of caudal peduncle at 2^nd^ dorsal-fin insertion 1.07 (1.16–1.40) times its width, 2.08 (1.43–1.48) times in dorsal–caudal space. Precaudal pits present; upper pit a deep, arcuate and crescentic depression; lower pit a much shallower crescentic depression.

**Fig 2:**
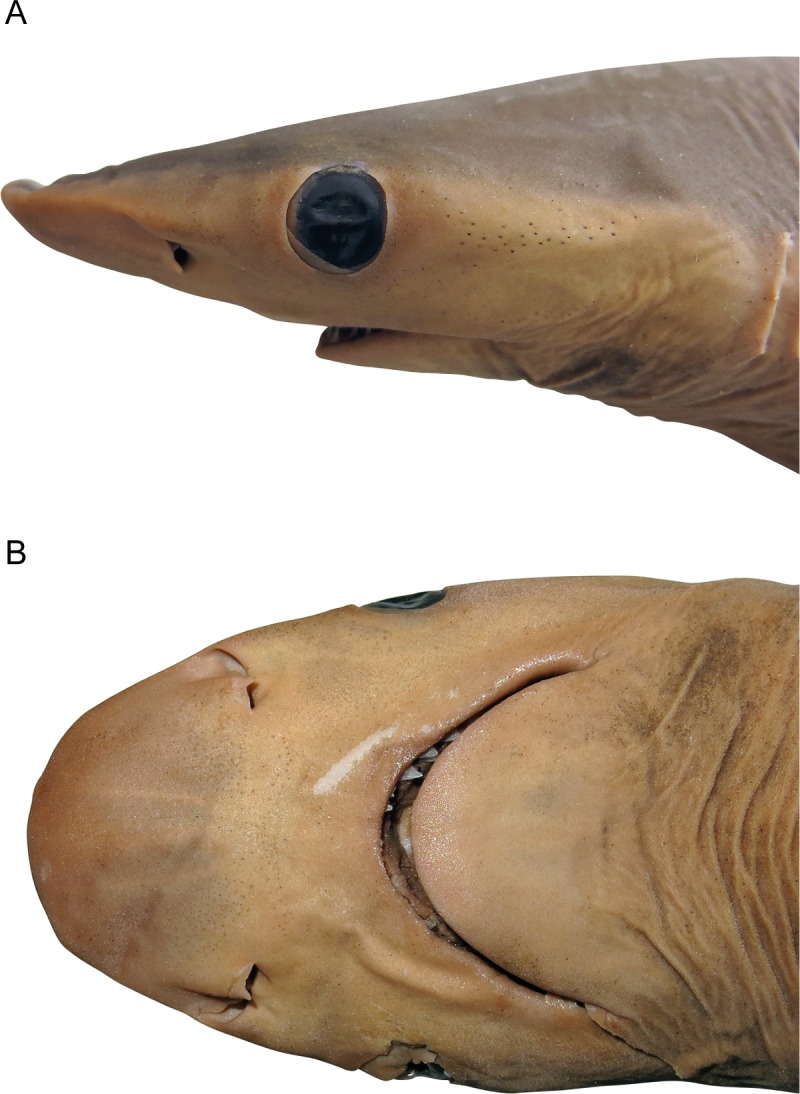
Head of *Carcharhinus obsolerus* sp. nov. (NMW 61463; Holotype). 433 mm TL female: A. lateral view; B. ventral view.

Head length to 5^th^ gill opening 0.76 (0.77) times in pectoral–pelvic space. Head narrow and slightly flattened, ellipsoidal-lenticular in shape in cross-section at eyes. Outline of head in lateral view undulated dorsally, nearly straight on snout, moderately convex above gills (Fig 2A); weakly convex ventrally along lower jaws and beneath gills. In dorsoventral view, head parabolic (Fig 2B); gill septa expanded slightly outwards. No distinctly enlarged hyomandibular pores adjacent to mouth corners. Snout moderately short, preoral snout length 1.07 (0.90–1.04) times mouth width; tip rounded in dorsoventral view and very weakly indented anterior to nostrils; snout bluntly pointed in lateral view, nearly straight above to weakly convex above and convex below. A narrow, longitudinal band of enlarged pores posterior to eye, almost entirely situated in the whitish ventral colouration (i.e. below the waterline).

External eye opening of fleshy orbit without anterior or posterior notches, circular in shape, with height 0.97 (1.06–1.08) in eye length. Eyes large, length 10.11 (8.11–10.01) in head length; situated laterally, with lower edges not crossing horizontal head rim in dorsal view; subocular ridges absent. Nictitating lower eyelids internal, with deep subocular pouches and secondary lower eyelids fused to upper eyelids. Spiracles absent. Fifth gill slit shortest, third and fourth gill slits largest, fifth about 0.75 (0.81–0.83) of height of third; height of third about 7.58 (8.31–11.01) in head length and 1.33 (0.91–0.98) times eye length. Gill slits upright, not oblique; margins of gill slits weakly concave. Gill filaments not visible from outside. Upper end of highest gill opening (third) just below level of upper margin of eye. Gill-raker papillae absent from gill arches. Nostrils strongly oblique, slit-like with large oval incurrent apertures; prominent triangular anterior nasal flaps with narrowly pointed tips, mesonarial flaps absent, small suboval excurrent apertures, posterior nasal flaps absent; well in front of mouth; width 2.44 (3.43–3.56) in internarial width, 1.01 (1.29–1.69) in eye length, 1.31 (1.23–1.33) in longest gill-opening.

Mouth moderately rounded and large; width 2.98 (2.70–2.84) in head length; mouth length 1.67 (1.65–1.80) in mouth width. Lips concealing teeth when mouth is closed. Tongue large, flat and broadly rounded, filling floor of mouth. Maxillary valve narrow, width much less than eye diameter, strongly papillose. No large buccal papillae on floor or roof of mouth behind maxillary valve. Palate, floor of mouth and gill arches covered with buccopharyngeal denticles. Labial furrows short, restricted to mouth corners, uppers 0.3 (0.5–0.6)% TL, lowers concealed by overlapping upper lip; anterior ends of uppers far behind eyes by distance of almost half of mouth width.

**Fig 3:**
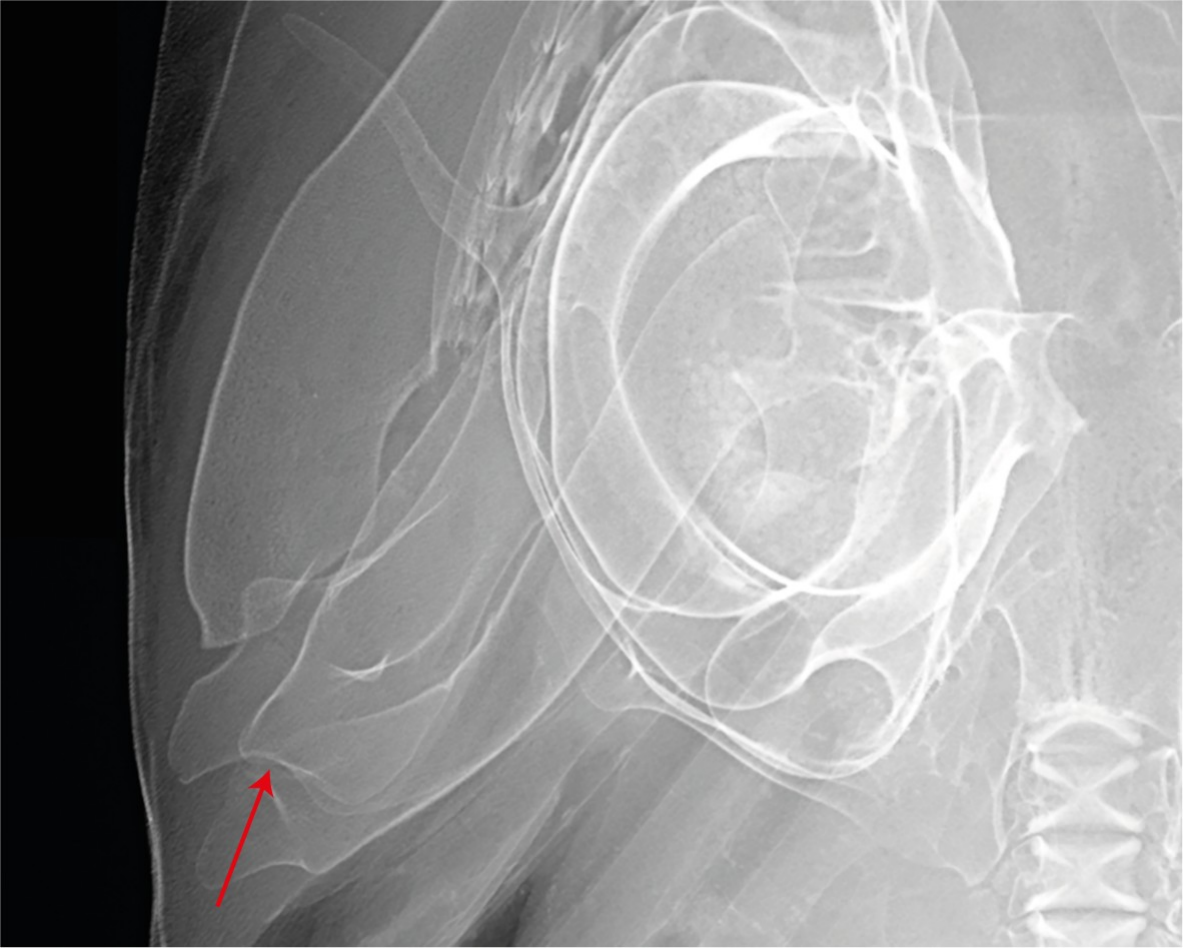
Post-mandibular indentation of Meckel’s cartilage in *Carcharhinus obsolerus* sp. nov. Radiograph of head of ANSP 77121, paratype; 433 mm TL female; arrow denotes indentation.

Dental meristics in the three known specimens range within 13–15 + 1 + 13–15 / 13–14 + 0–1 + 13–14, with the holotype having 14 + 1 + 14 / 14 + 1 + 14. Pronounced dignathic heterodonty between upper and lower jaws. Upper teeth broadly compressed and blade-like with semi-oblique to distally notched, oblique crowns and even labial surfaces. Lower teeth with narrow, erect, triangular crowns anteriorly, becoming oblique with slightly convex labial surfaces laterally. Post-mandibular indentation of Meckel’s cartilage elongated and shallow (Fig 3).

**Fig 4:**
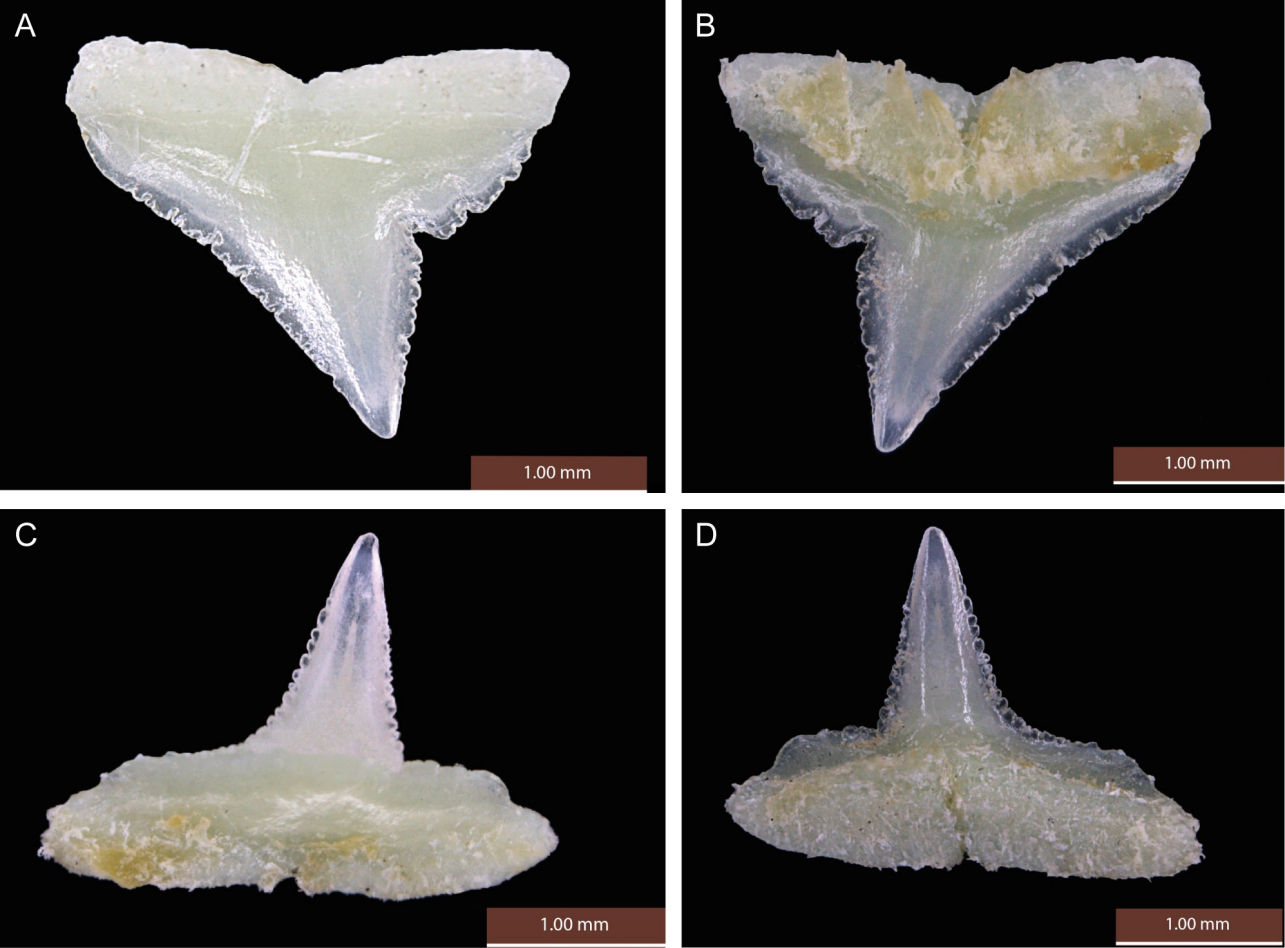
Teeth of *Carcharhinus obsolerus* sp. nov (ANSP 77121, paratype). 433 mm TL female; 4^th^ anterolateral tooth from left side of jaw: A. upper tooth labial view; B. upper tooth lingual view; C. lower tooth labial view; D. lower tooth lingual view.

**Fig 5:**
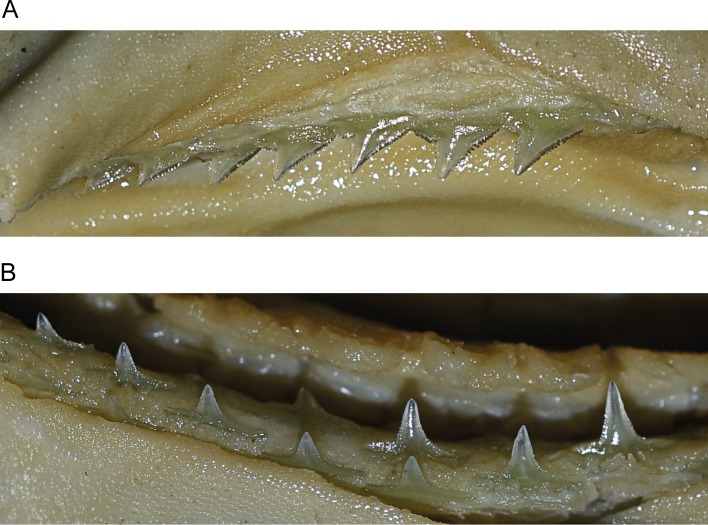
*In situ* teeth of *Carcharhinus obsolerus* sp. nov. (ANSP 77121, paratype). 370 mm TL female: A. upper teeth; B. lower teeth.

Upper jaw gradient monognathic heterodonty present in all tooth groups; one small, well-developed symphysial tooth present. Anterior teeth (Figs 4A, 4B and 5A) narrow with somewhat linear lower distal crown margins and weekly notched basal margins; mesial crown edges somewhat linear, not convex with apical portion of crowns slightly reflexed; serrations coarser basally and weaker apically. Anterolateral files with considerably more oblique crowns; distal margins notched with strong basal/apical bifurcation of crown; basal margins more coarsely serrated, no enlarged distal serrae present and serrations gradient from outer basal margins to crown notches; lower portion of crowns noticeably serrated, decreasing but present apically; mesial margins linear, not convex with apical portion of crowns slightly recurved mesially; serrations slightly coarser basally, decreasing but present apically. Lateroposterior files with considerably more oblique crowns than laterals; distal margins deeply notched; serrations coarser basally; mesial margins slightly more posteriorly arcuate and lacking mesial recurvature apically.

Lower jaw gradient monognathic heterodonty present but weak in all tooth groups; one small, well-developed symphysial tooth present. Anterior teeth (Figs 4C, 4D and 5B) with narrow, erect, triangular cusps, moderate in height and not noticeably elongated with strongly acuminate apical portions; distal edge of crowns slightly angular and weakly notched; mesial edges of crowns somewhat concave with apical portion slightly recurved mesially; contour of roots not crescentic or arched with evident root lobes; distal and mesial cusp edges moderately serrated; coarser basally. Lateral files more noticeably notched with slightly more angular distal margins; crown feet coarsely serrated on both distal and mesial margins; slightly heavier distally; lower crown portion with slightly acuminate apical margins and moderately serrated edges, decreasing but present apically. Lateroposterior files increasingly angular with heavier distal notches; distal basal margins heavily serrated, less so on mesial margins; lower crown portions moderately serrated, decreasing but present apically; lower cusp portion slightly crescentic with apical portions recurved mesially.

**Fig 6:**
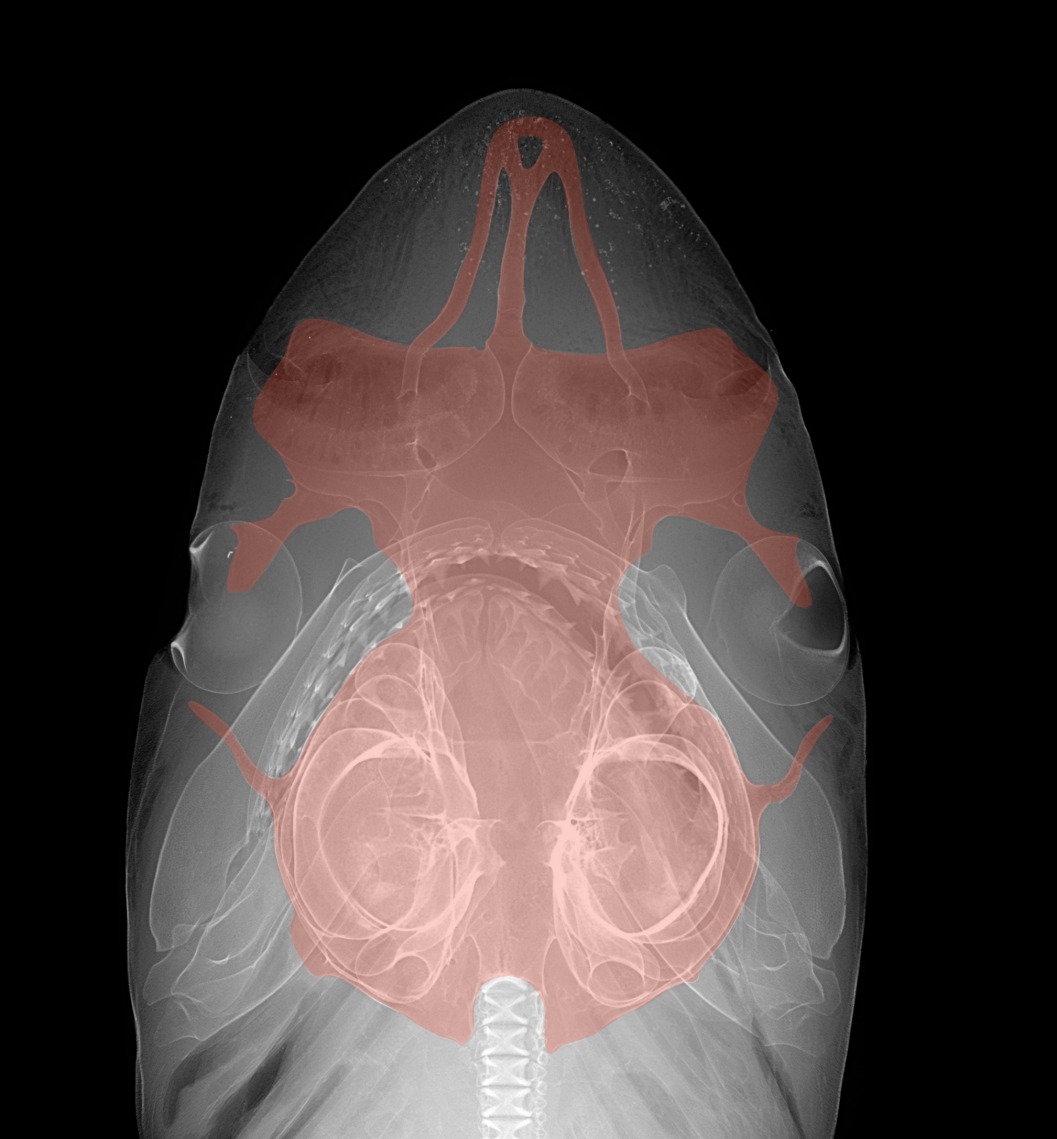
Digital radiograph of the head of *Carcharhinus obsolerus* sp. nov. (ANSP 77121, paratype). 370 mm TL female; chondrocranium highlighted in red.

Rostral cartilages moderately slender, not hypercalcified, rostral fenestrae relatively large, rostral tip truncate, not pointed; nasal capsules broad, anterior margins nearly straight; anterior fontanelle moderately expanded, posterior border with a distinct indentation centrally; preorbital processes large, somewhat triangular at tip, relatively narrow based; postorbital processes long and slender; orbits large (Fig 6).

**Fig 7:**
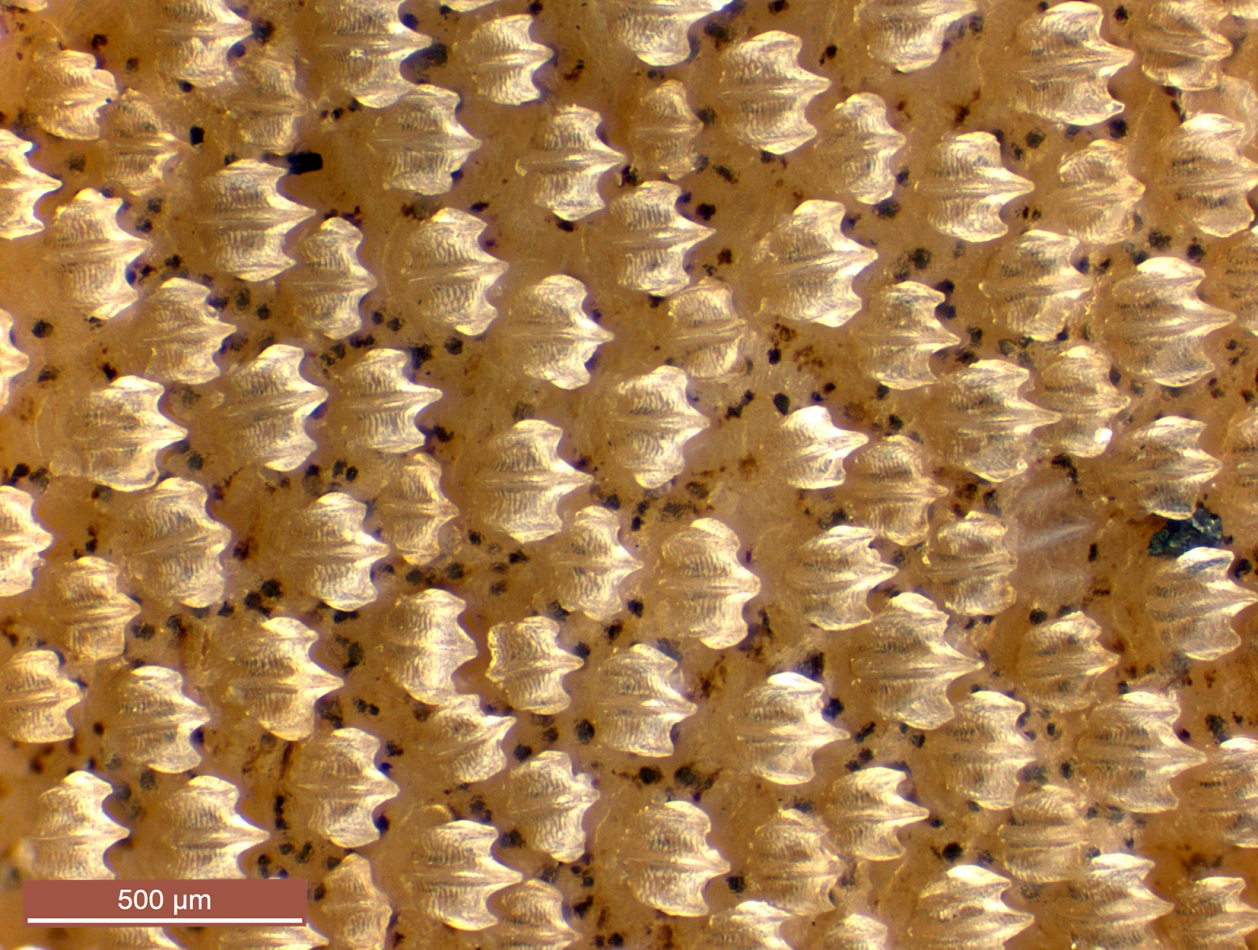
Lateral trunk denticles of *Carcharhinus obsolerus* sp. nov. (NMW 61463, holotype). 433 mm TL female.

Lateral trunk denticles small, slightly imbricate, broad, tricuspid (Fig 7); crowns usually slightly wider than long (sparser smaller denticles slightly longer than wide), with 3 prominent longitudinal ridges (medial ridge slightly stronger and more pronounced) that extend entire length of crown onto cusps; medial cusp short but strong, much shorter than rest of crown, flanked by a pair of slightly shorter lateral cusps.

Pectoral fins short and relatively broad, weakly falcate; anterior margin moderately convex, apices narrowly rounded; posterior margin very weakly concave; free rear tip moderately rounded to somewhat angular, inner margin weakly convex; base broad about 57 (55–59)% of fin length; length from origin to rear tip 1.26 (1.20–1.24) times anterior margin length; larger in area to first dorsal fin; origin under fourth gill slit; fin apex posterior to free rear tip when fin is elevated and adpressed to body.

Pelvic fins small, triangular and not falcate; length of anterior margin 0.50 (0.41–0.42) of pectoral-fin anterior margin; area about 1.5 times that of anal fin; anterior margin nearly straight; apex rounded angular; posterior margin nearly straight; free rear tip bluntly rounded, inner margin nearly straight.

First dorsal fin relatively small, moderately long-based, broad and triangular, not falcate; anterior margin weakly convex; apex moderately rounded; posterior margin distally straight and basally moderately concave; free rear tip acutely pointed, inner margin nearly straight; origin situated posterior to pectoral-fin insertion by about a third of the pectoral-fin inner margin length, midpoint of base 1.72 (1.93–2.68) times closer to pectoral insertions than pelvic origins; free rear tip anterior to pelvic-fin origins by about an eye length; posterior margin arching posteroventrally from apex, then abruptly to near free tip; insertion posterior to dorsal-fin apex. First dorsal fin base 2.24 (1.91–1.96) in interdorsal space, 2.50 (2.51–2.66) in dorsal caudal margin; height 1.21 (1.13–1.34) in base; inner margin 2.07 (1.68–1.89) in height, 2.52 (1.90–2.54) in base.

**Fig 8:**
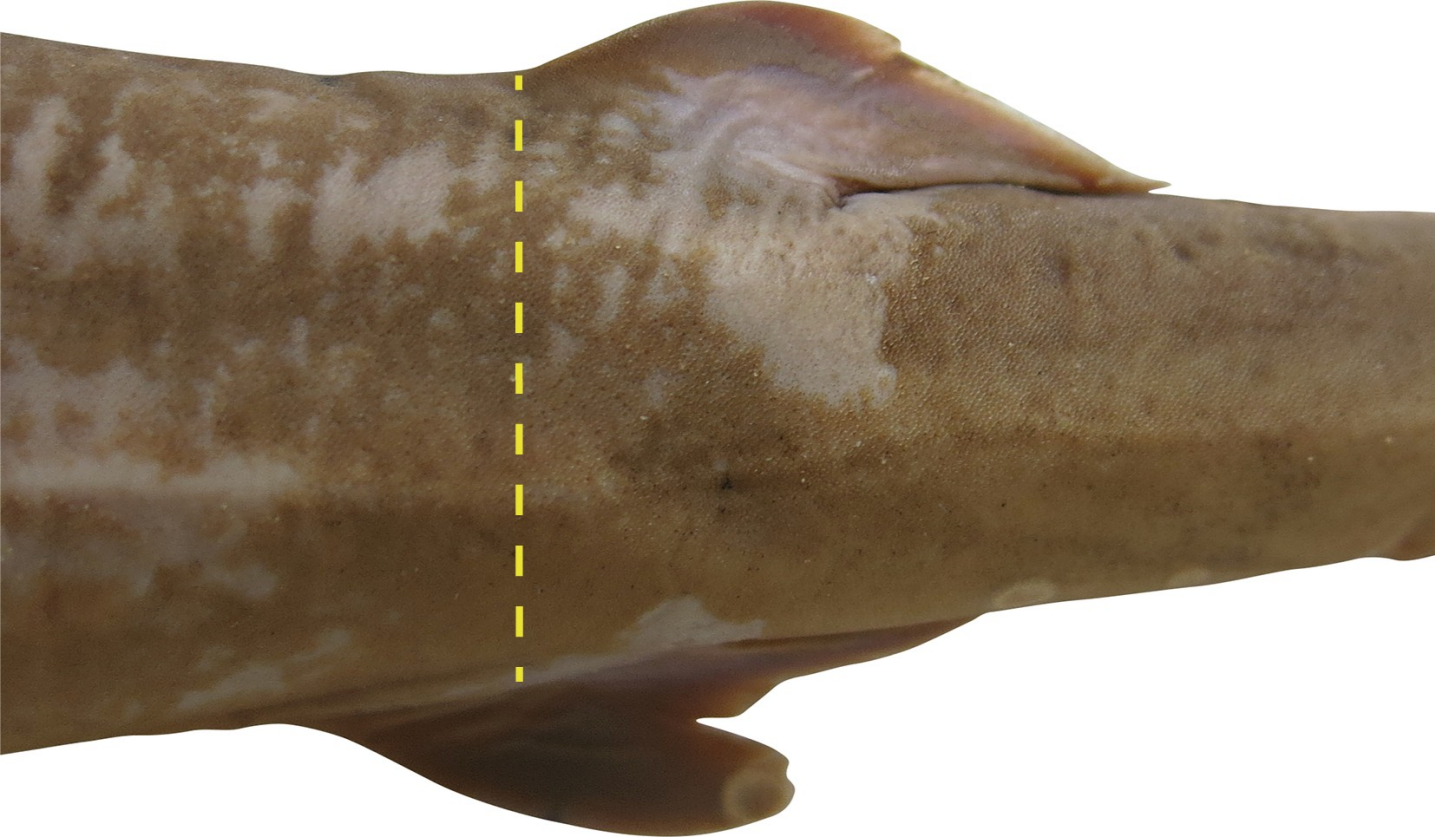
Alignment of second dorsal fin in relation to anal fin of *Carcharhinus obsolerus* sp. nov. NMW 61463, holotype (433 mm TL female); yellow dashed line indicates alignment of origin of second dorsal fin with anal-fin midbase.

Second dorsal fin very small and low, subtriangular; height 4.16 (3.21–4.45) in first dorsal-fin height, base 2.52 (2.78–3.07) in first dorsal-fin base; anterior margin weakly convex; apex rounded; posterior margin weakly concave; free rear tip long, acutely pointed, inner margin nearly straight; origin opposite anal-fin midbase (Fig 8); rear tip well behind anal-fin free rear tip, in front of upper caudal-fin origin by 1.36 (0.43–1.00) times its inner margin; posterior margin directed strongly posteroventrally from apex; insertion about opposite fin apex. Second dorsal fin base 2.02 (1.56–1.82) in dorsal–caudal space; height 2.01 (1.40–1.81) in base; inner margin 1.71 (1.28–1.98) times height, 1.17 (0.91–1.09) in base.

Anal fin apically narrow and strongly falcate; slightly larger than second dorsal fin; height 1.49 (1.17–1.25) times second dorsal-fin height, base length 1.21 (1.11–1.17) times second dorsal-fin base; anterior margin moderately convex; apex narrowly rounded; posterior margin deeply notched at slightly less than a right angle; free rear tip acutely pointed, inner margin nearly straight; origin well anterior to second dorsal-fin origin; insertion about level with second dorsal-fin midbase, anterior to fin apex; free rear tip in front of lower caudal-fin origin by a length slightly more than its inner margin length; posterior margin slanting anterodorsally and then abruptly posterodorsally. Anal-fin base expanded anteriorly as very short preanal ridges (obscure), less than a quarter length of rest of base. Anal-fin base 1.77 (1.47–1.55) in anal–caudal space; height 1.64 (1.40–1.60) in base; inner margin 1.18 (1.28–1.52) times height, 1.38 (1.05–1.09) in base.

Caudal fin narrow-lobed and asymmetrical, with short terminal lobe and prominent but moderately long, non-falcate ventral lobe; dorsal caudal margin proximally and distally convex, and slightly concave just anterior to subterminal notch, with prominent lateral undulations; preventral margin moderately convex, tip of ventral caudal-fin lobe narrowly rounded; lower postventral margin weakly concave; upper postventral margin straight; subterminal notch a narrow, deep slot; subterminal margin nearly straight; terminal margin irregular and weakly concave, lobe formed by these margins angular, tip of tail narrowly rounded. Length of dorsal caudal margin 2.98 (2.35–2.54) in precaudal length, preventral caudal margin 2.28 (2.35–2.66) in dorsal caudal margin, terminal lobe from caudal tip to subterminal notch about 3.15 (3.19–3.62) in dorsal caudal margin, subterminal margin length 1.57 (2.14–2.24) in terminal margin.

Counts of total vertebral centra (TC) 120 (117–118), precaudal centra (PC) 54 (58), monospondylous precaudal (MP) centra 36 (39–40), diplospondylous precaudal (DP) centra 18 (18–19), diplospondylous caudal (DC) centra 66 (59–60); MP centra 30 (34.2)%, DP centra 15 (15.4–16.7)%, and DC centra 55 (49.1–50.4)% of TC centra. Ratios of DP/MP centra 0.50 (0.45–0.49), DC/MP centra 1.83 (1.44–1.48).

#### Colour

In preservative: dorsal surface of head, trunk and tail grey, graduating to pale ventral colouration on midlateral surfaces. Demarcation between light and dark surfaces of head strong (light ventral colour just visible in dorsoventral view of head), extending along lateral angle of the snout anteriorly to level of nostrils, extending dorsoposteriorly to level of upper margin of eye; then extending very gradually ventroposteriorly to about upper edge of first gill slit; waterline more diffuse over gills, almost at level of upper margins; gill slit membranes mostly pale; margins of gill slits narrowly pale edged. Demarcation between dorsal and ventral coloration becoming diffuse above pectoral fins; extending diffusely along abdomen and tail at about midlateral level; pale area continuing onto base of caudal fin. Fins without any obvious dark or light markings. First dorsal fin grey, lower margin of free tip pale. Second dorsal grey, posterior margin slightly paler. Anal fin slightly paler grey. Caudal fin grey, terminal and ventral lobes slightly darker. Pectoral fins grey on both surfaces, posterior margins diffusely pale-edged. Pelvic fins pale grey, much paler on ventral surfaces basally. Eyes blackish; nictitating membrane pale.

### Size

Only known from the three type specimens, a late-term embryo 339 mm TL and two juvenile females 370 and 433 mm TL. Size at birth likely close to 340 mm TL, since late-term embryo was fully developed and 370 mm TL juvenile had a faint umbilical scar.

### Distribution

Uncertain; collection records indicate southern South China Sea (Gulf of Thailand, Vietnam, Malaysian Borneo).

### Etymology

The specific name is Latin for ‘extinct’ (*obsolerus*) in allusion to the fact that the species has not been recorded in many decades. Proposed English vernacular name: Lost Shark.

## Discussion

*Carcharhinus obsolerus* can be readily separated from most of its congeners by the relative position of the second dorsal and anal fins, and its low vertebral count. The second dorsal-fin origin of *C*. *obsolerus* is about level with the mid anal-fin base, a feature shared by *C*. *borneensis*, *C*. *cerdale*, *C*. *macloti*, and *C*. *porosus*, and occasionally in *C*. *hemiodon* [19, 24]. While the second dorsal-fin origin of *C*. *sealei* is often posterior to its anal-fin origin, it is never level with the mid anal-fin base [12]. In contrast, the remaining 29 species in this genus have the origin of the second dorsal fin either level with, or anterior to the origin of the anal fin. The relative positions of the second dorsal and anal fin do not vary ontogenetically based on other closely related species [12, 21]. Thus, this character is considered a key character for separating these taxa.

**Fig 9:**
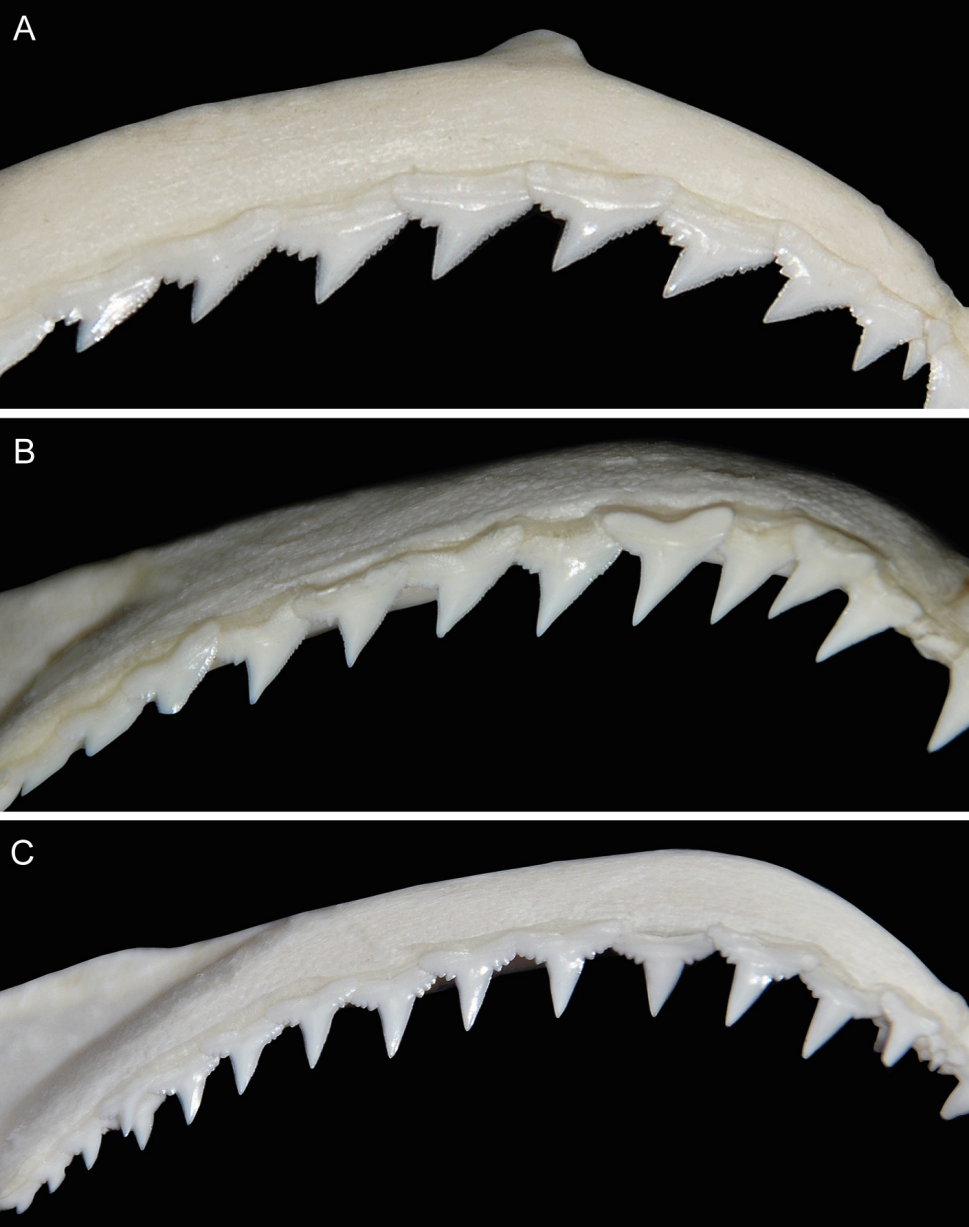
Upper jaw dentition in the three most similar *Carcharhinus* species. A. *Carcharhinus borneensis* (PMH244-1, mature female 661 mm TL); B. *Carcharhinus cerdale* (PMH329-01, juvenile male 887 mm TL; left side of jaw with image reversed); C. *Carcharhinus macloti* (PMH057-15, mature female 1090 mm TL).

**Fig 10:**
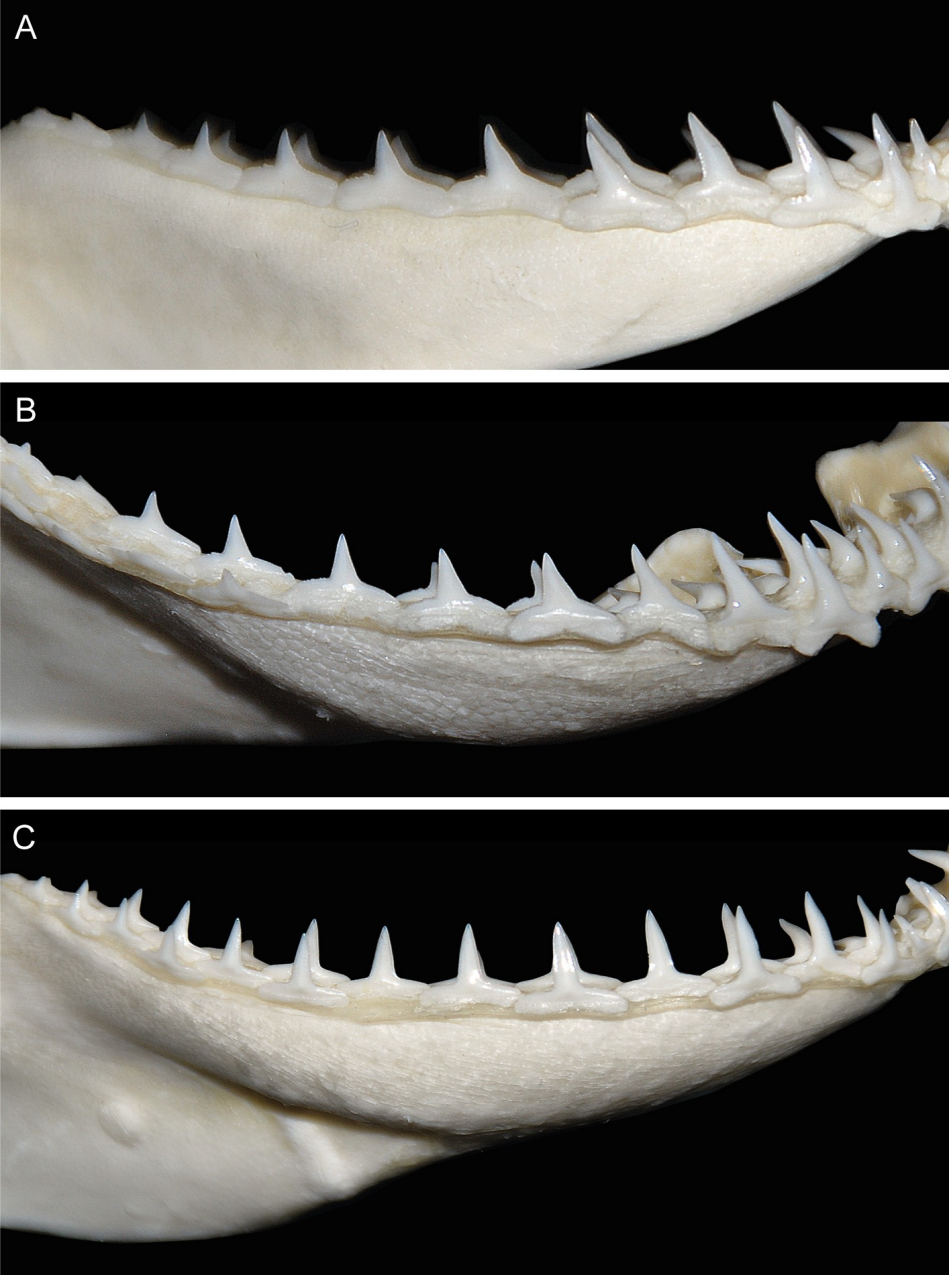
Lower jaw dentition in the three most similar *Carcharhinus* species. A. *Carcharhinus borneensis* (PMH244-1, mature female 661 mm TL); B. *Carcharhinus cerdale* (PMH329-01, juvenile male 887 mm TL; left side of jaw with image reversed); C. *Carcharhinus macloti* (PMH057-15, mature female 1090 mm TL).

The new species has one of the lowest vertebral counts within the genus. Precaudal and total vertebral counts of *C*. *obsolerus* are 54–58 and 117–120, respectively. Note that while the same vertebral counts were obtained as [19] for the holotype, the precaudal count for ANSP 76859 was one more than [19]. Also, we counted an additional precaudal centrum and one less caudal centrum for ANSP 77121. Only three other species have both precaudal and total counts overlapping: *C*. *borneensis* (57–63 and 117–121, respectively), *C*. *porosus* (41–57 and 96–122, respectively), and *C*. *tjutjot* (55–63 and 113–129, respectively) (S2 Table). *Carcharhinus* species with low but non-overlapping vertebral counts are *C*. *cerdale* (62–67 and 129–135, respectively), *C*. *coatesi* (67–76 and 134–147 respectively), *C*. *dussumieri* (62–68 and 123–138, respectively), *C*. *hemiodon* (69–71 and 147–155 respectively), *C*. *fitzroyensis* (58 and 125, respectively), and *C*. *macloti* (59–71 and 135–154, respectively). All other *Carcharhinus* species have precaudal and total vertebral counts in excess of 66 and 150, respectively (S1 Table). Based on the relative positions of the second dorsal and anal fins, and its low vertebral count, *C*. *obsolerus* is readily distinguished from all its congeners except *C*. *borneensis* and *C*. *porosus*. Tooth counts and morphology of *Carcharhinus obsolerus* are closest to *C*. *borneensis*, *C*. *cerdale/porosus* and *C*. *macloti* (S1 Table, Figs 9 and 10). *Carcharhinus macloti* differs from *C*. *obsolerus* in having entirely smooth-edged lower crown portions in both the upper and lower jaw teeth (Figs 9C and 10C) compared to serrations on all crown margins in both jaws. Female specimens of *C*. *obsolerus* also have oblique crowns in the lower jaw teeth vs. perpendicular crowns in *C*. *macloti* (Fig 10C).

*Carcharhinus obsolerus* differs from *C*. *borneensis* in having a higher tooth count (upper teeth 27–31 vs. 23–26; lower teeth 26–29 vs. 23–25); tooth morphology, concave mesial margins and cusp recurvature in the upper and lower anterolateral teeth vs. more lineal mesial margins that lack cusp recurvature in the apical portion in *C*. *borneensis*, very large and coarse serrations basally (but non-lobate) present on the distal margins of the upper teeth vs. large and lobate basal serrae in *C*. *borneensis* (Fig 9A) and *C*. *macloti* (Fig 9C); the absence of enlarged hyomandibular pores lateral to each mouth corner (vs. 5–12 enlarged pores alongside each mouth corner); and in the following morphological characters (based on morphometric data for all sizes of *C*. *borneensis* in [21]): wider head (interorbital space 11.2–12.0 vs. 8.9–10.5% TL), slightly larger eyes (eye length 2.4–2.9 vs. 1.6–2.5% TL, 8.1–10.1 vs. 10.0–15.1 times in head length), longer pelvic-fin posterior margin (5.2–5.6 vs. 3.5–4.9% TL), and slightly larger pectoral fins (anterior margin 14.6–16.8 vs. 10.9–14.8% TL, height 12.4–13.7 vs. 9.6–12.8% TL, posterior margin 10.9–12.2 vs. 7.9–11.6% TL). *Carcharhinus obsolerus* also differs from *C*. *borneensis* in cranial morphology in having broader nasal capsules with straight anterior margins (vs. angled anterior margins angled, see Fig 19.10H in [16]).

Given the potential for ontogenetic variation in morphology further comparisons with were made with four similarly-sized *C*. *borneensis* (341–373 mm TL). The *C*. *obsolerus* types (339–433 mm TL) differed from these specimens in the following characters: shorter prenarial snout (horizontal prenarial length 4.8–5.7 vs. 5.8–6.0% TL; snout tip to inner nostril 5.5–6.7 vs. 6.8–7.0% TL, 1.9–2.7 vs. 2.8–3.6 times eye length), slightly taller first dorsal fin (height 8.2–9.9 vs. 7.5–8.3% TL), longer upper postventral caudal margin (11.9–15.5 vs. 10.4–10.8% TL), shorter caudal subterminal margin (3.0–3.8 vs. 4.2–4.9% TL), pelvic fins slightly more separated from first dorsal fin (first dorsal-fin base midpoint to pelvic-fin origin 11.5–13.4 vs. 9.8–10.8% TL), and second dorsal-fin insertion closer to level of anal-fin insertion (0.8–1.3 vs. 1.6–2.2% TL).

*Carcharhinus obsolerus* differs from *C*. *porosus* in tooth morphology: shorter cusps and somewhat linear basal structures vs. more elongated cusps in the anterior and lateral files (rows) of the lower jaw, as well as more deeply arched root margins in *C*. *porosus* as well as *C*. *cerdale* (Fig 9B); and in the following morphological characters (based on morphometric data for similar-sized individuals in [19]): preanal length (57.5–60.7 vs. 60.4–62.8% TL), mouth width (8.3–8.9 vs. 7.0–7.8% TL, 7.9–9.0 vs. 9.5–10.9 in precaudal length, 6.5–7.3 vs. 7.7–8.8 in preanal length), eye size (eye length 2.4–2.9 vs. 2.2–2.5% TL), and pectoral fin size (base length 6.1–6.9 vs. 5.2–6.0% TL, anterior margin 14.6–16.8 vs. 13.2–14.9% TL). *Carcharhinus obsolerus* also differs from *C*. *porosus* in having a truncate vs. pointed rostral tip (see Fig 19.10G in [16]).

**Fig 11:**
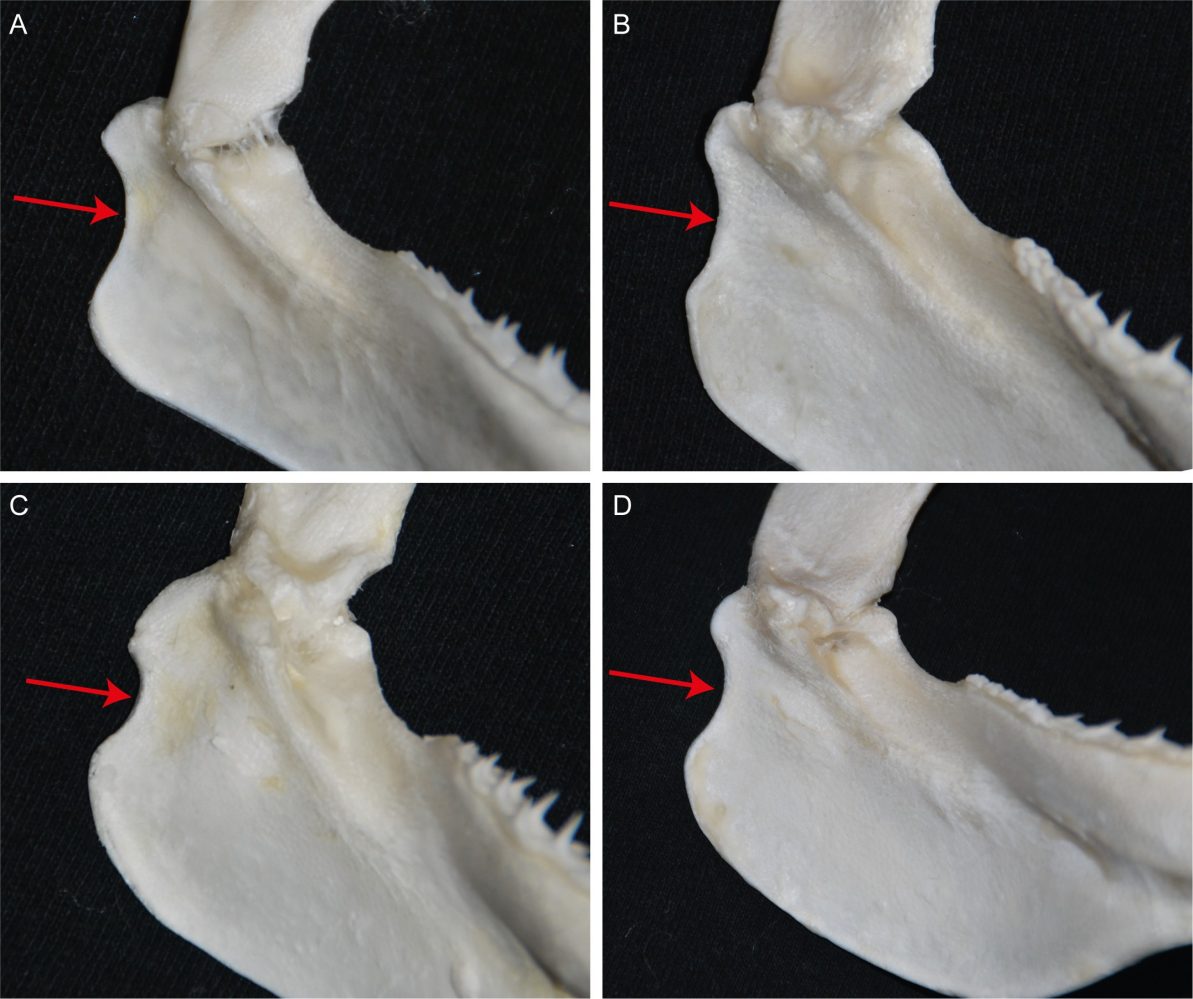
Post-mandibular indentation of Meckel’s cartilage. Arrow denotes indentation: A. *Carcharhinus borneensis* (PMH 244–1, female 661mm TL); B. *Carcharhinus cerdale* (PMH 329–01, male 887 mm TL); C. *Carcharhinus macloti* (PMH 057–14, male 970 mm TL; D. *Carcharhinus porosus* (PMH 121–01, female 1085 mm TL).

Finally, in *Carcharhinus obsolerus* and *C*. *borneensis*, the indentation in the posterior edge of the mandibular plate (rear Meckel’s cartilage) is elongated and shallow (Figs 3 and 11A). In contrast, this indentation is shorter and deeper in *C*. *cerdale*, *C*. *porosus* and *C*. *macloti* (Fig 11B–11D).

Although [19] considered the three specimens of *C*. *obsolerus* conspecific with *C*. *porosus*, he stated that decision was “despite the seemingly improbable distribution for a small, essentially tropical, littoral shark” and that adult specimens are required. In contrast, [15] and subsequently [16] considered it a distinct, undescribed species, based on morphometrics and cranial morphology. However, due to the broad scope of [15, 16], these differences were not specified. More recently, [18] considered these three specimens as questionably an undescribed species but the data provided was largely taken from [19] with no additional insights provided. The decision to allocate these three specimens to a new taxon in this paper was not purely a different interpretation to the work of [19]. An important consideration when examining the *C*. *porosus* treatment in [19] is that it represents two taxa, *C*. *porosus* and *C*. *cerdale* which was subsequently resurrected as a valid species separate from *C*. *porosus* by [14].

The taxonomic decision made in this study to formally describe these three specimens as a new species carefully considered the information in all of the aforementioned publications and new insights gained from examination of these specimens. The need for obtaining adult specimens alluded to by [19] was an appropriate conclusion to make at that time. Prior to 1982, there had been very few detailed biodiversity surveys or investigation of catch composition in coastal fisheries in the South-east Asian region. Thus the lack of adult records could easily be linked to inadequate collecting in the area it occurred. However, in recent decades there has been numerous detailed biodiversity and fisheries surveys in the South-east Asian region which have revealed no further specimens of this species. This is an important consideration as it changes the context of the conclusions made by [19] and those made in this study. The lack of additional specimens despite the numerous comprehensive surveys in the species known range possibly suggest that this species is extinct.

Morphological differences between *C*. *obsolerus* and *C*. *porosus*, from the Western Atlantic only, found in this study are considered compelling enough to consider them separate species. This is strengthened by the cranial differences found as well as the detailed dental comparisons made (see S2 Table).

### Distribution

The distribution of *Carcharhinus obsolerus* is uncertain. Given that this species has not been seen in many decades, a better understanding of the distribution of this species is unlikely unless archaeological or paleontological records are found. While Baram in Sarawak is likely an accurate collection locality, both Bangkok and Ho Chi Minh City specimens may have been caught in other South-east Asian locations and brought into these cities where bigger markets exist. Thus, there is a possibility it had a much more restricted distribution than the three known specimens allude to, but it cannot be ruled out that it had a wider distribution in the South-east Asian region.

### Conservation implications

*Carcharhinus obsolerus* n. sp. has not been collected or identified in the field since 1934. Extensive fish market surveys conducted during the past two decades have found no recent specimens of this species. It was not recorded in surveys of chondrichthyan fishes around Borneo, including in Baram where one of the paratypes were collected [34], or during market surveys in areas adjacent to its range, including Indonesia [35] and the Philippines [17, 36].

While the lack of contemporary records of *C*. *obsolerus* is of great concern for its conservation status, there is a possibility that further specimens are identified in the future. The rediscovery of *C*. *borneensis* in 2004 in Sarawak, Borneo [21] demonstrates that “lost” marine species may still be extant despite a long absence of records. That species had not been recorded since 1937.

The lack of records restricts an accurate assessment of the life history parameters and habitat requirements of *C*. *obsolerus*. Species of the *C*. *porosus* subgroup are relatively small-bodied, but available biological data implies limited productivity. *Carcharhinus porosus* reaches sexual maturity at 6 years, with a longevity of 12 years, and a slow growth rate (*k* <0.10) [37]. Litter size in that species ranges 2–9, with a gestation period of ~1 year [38]. Similarly, a gravid female *C*. *borneensis* contained 6 pups [21], also suggesting limited fecundity.

Species of the *C*. *porosus* subgroup generally occur in shallow coastal waters [33], which are accessible to fisheries. The areas from which the three known *C*. *obsolerus* specimens were collected, Thailand, Vietnam, and Borneo, are subject to intense, largely unregulated coastal fisheries. The combination of limited biological productivity, and occurrence in shallow coastal habitats exposed to intense fishing, elevates extinction risk for this group, including *C*. *obsolerus*.

## Supporting information

S1 Table Vertebral and tooth count summaries for the genus *Carcharhinus*. Ranges for number of precaudal and total centra, and upper and lower teeth (with number of specimens included in range in parantheses) for members of the genus *Carcharhinus*. Species groupings follow a combination of information provided in [19] and molecular results in [30]; note these are only provisional subgroupings pending a more detailed phylogenetic revision of this genus.
(XLSX)

S2 Table Comparison of the tooth and jaw morphology. Key tooth and jaw characters useful for distinguishing between *Carcharhinus obsolerus*, *Carcharhinus cerdale/porosus*, *Carcharhinus borneensis* and *Carcharhinus macloti*.
(DOCX)
