# Supplementary material for: Lost before found: A new species of whaler shark Carcharhinus obsolerus from the Western Central Pacific known only from historic records
Source: PLoS One. 2019 Jan 2;14(1):e0209387. doi: 10.1371/journal.pone.0209387 (PMC6314596; doi:10.1371/journal.pone.0209387)
Supplement: S2 Table — Key tooth and jaw characters useful for distinguishing between Carcharhinus obsolerus, Carcharhinus cerdale/porosus, Carcharhinus borneensis and Carcharhinus macloti. (DOCX) [file pone.0209387.s002.docx]

**S2 Table. Comparison of the tooth and jaw morphology.** Key tooth and jaw characters useful for distinguishing between *Carcharhinus obsolerus*, *Carcharhinus cerdale/porosus*, *Carcharhinus borneensis* and *Carcharhinus macloti*.

| **Character** | ***C. obsolerus*** | ***C. borneensis*** | ***C. cerdale/porosus*** | ***C. macloti*** |
| --- | --- | --- | --- | --- |
| Tooth count (upper/lower) | 27–31 / 26–29 | 23–26 / 23–25 | 27–31 / 26–30 | 29–33 / 26–31 |
| Tooth edges | Serrated | Serrated | Serrated | Smooth |
| Sexual dimorphism | Unknown, only females examined | Strongly evident. Males with very narrow, oblique cusps in upper and lower teeth | Not strongly evident. Males with only slightly narrower upper teeth | Strongly evident. Males with very narrow, oblique cusps in upper and lower teeth. |
| Upper teeth | Broadly triangular; anterior teeth not elongated with more linear root structure | Broadly triangular in females, narrower in males; anterior teeth not elongated with more linear root structure | Broadly triangular; anterior teeth elongated with narrow, somewhat concave and arched root structure | Very narrow and triangular; anterior teeth not elongated with more linear root structure |
| Mesial margin of upper anterolateral teeth | Slightly crescentic with apical margin recurved slightly mesially | Linear without apical margin recurved mesially | Linear without apical margin recurved mesially | Linear without apical margin recurved mesially |
| Basal margins of upper teeth | Coarsely serrated but no enlarged, angularly lobate serrae on distal portion | Coarsely serrated; females with 2–3 large, angularly lobate serrae on distal edge; males usually with 3–4 | Coarsely serrated but no enlarged, angularly lobate serrae on distal portion | Without serrations but with 1–3 enlarged, angularly lobate serrae on distal portion |
| Lower anterior teeth | Without elongated cusps and somewhat angular distally; somewhat concave mesial margins and apical portions noticeably recurved mesially; cusps moderately serrated; finely serrated basally | Without elongated cusps but somewhat angular distally; somewhat convex mesial margins and apical portions directed distally; cusps moderately serrated; finely serrated basally | With elongated cusps, somewhat angular distally; linear mesial margins and apical margins only slightly recurved mesially; cusps very finely serrated; serrations absent basally | Without elongated cusps, females with straight cusps, males with oblique cusps, not angular; convex mesial margins with apical portions directed distally; cusps entirely smooth |
| Root structure of lower anterior teeth | Linear and not concave and arched | Linear and not concave | Somewhat concave and arched | Linear and not concave |
| Cusps of lower lateral teeth | Coarsely serrated; not elongated and somewhat vertical | Moderately serrated; not elongated and noticeably angled distally | Finely serrated; elongated and somewhat angled distally | Entirely smooth; not angular, vertical in females and oblique in males |
| Post-mandibular indentation of lower jaw | Elongate and shallow | Elongate and shallow | Shorter and deep | Shorter and deep |
